# Gut microbiota mediates the inhibition of lymphopoiesis in dietary-restricted mice by suppressing glycolysis

**DOI:** 10.1080/19490976.2022.2117509

**Published:** 2022-09-01

**Authors:** Si Tao, Yiting Wang, Chenghui Yu, Rongrong Qiu, Yanjun Jiang, Jie Jia, Zhendong Tao, Liu Zhang, Bing Zou, Duozhuang Tang

**Affiliations:** a Jiangxi Key Laboratory of Clinical and Translational Cancer Research, Department of Oncology, The Second Affiliated Hospital of Nanchang University, Jiangxi, China; b Department of Oncology, The Second Affiliated Hospital of Nanchang University, Jiangxi, China; c Department of Hematology, The Second Affiliated Hospital of Nanchang University, Jiangxi, China; d Department of Medical Laboratory Medicine, Jiangxi Province Hospital of Integrated Chinese & Western Medicine, Jiangxi, China; e Intensive Care Unit, Peking University People’s Hospital, Beijing, China

**Keywords:** Dietary restriction, gut microbiota, lymphopoiesis, glycolysis, butyrate

## Abstract

Dietary restriction (DR) is one of the most robust interventions shown to extend health-span and remains on the forefront of anti-aging intervention studies, though conflicting results have been shown on its effect on lifespan both in rodents and primates. The severe inhibitory effects on the lymphoid lineage by DR remains one of its major negative downsides which reduces its overall beneficial effects on organismal health. Yet, the underlying mechanism of how DR suppresses the lymphoid system remains to be explored. Here, we show that antibiotic ablation of gut microbiota significantly rescued the inhibition of lymphopoiesis by DR. Interestingly, glycolysis in lymphocytes was significantly down-regulated in DR mice and pharmacological inhibition of glycolysis reverted this rescue effect of lymphopoiesis in DR mice with ablated gut microbiota. Furthermore, DR remarkably reconstructed gut microbiota with a significant increase in butyrate-producing bacterial taxa and in expression of *But*, a key gene involved in butyrate synthesis. Moreover, supplemental butyrate feeding in AL mice suppressed glycolysis in lymphoid cells and mimicked the inhibition of lymphopoiesis in AL mice. Together, our study reveals that gut microbiota mediates the inhibition on lymphopoiesis via down-regulation of glycolysis under DR conditions, which is associated with increased butyrate-synthesis. Our study uncovered a candidate that could potentially be targeted for ameliorating the negative effects of DR on lymphopoiesis, and therefore may have important implications for the wider application of DR and promoting healthy aging.

## Introduction

Under the increasing pressure of an aging population, studies have shown growing interest regarding the prevention and amelioration of aging related pathologies and diseases. Dietary restriction (DR), a 20%–40% reduction in daily food intake without leading to malnutrition,^1^ has been intensively studied since 1935, when it was shown to double the lifespan of experimental rats, and was proven to prevent or retard the development of various aging-related pathologies and diseases, such as cancer, diabetes and cardiovascular diseases.^1–6^ Therefore, DR holds a promising hope to help release the heavy medical burden of aging societies. However, despite the unambiguously documented beneficial effects of DR on health parameters, there are conflicting results about the improvement on overall survival and lifespan by DR in primates^7–9^ and different mouse strains.^10–12^ Previously we have shown that DR is a double-edged sword to the hematopoietic system, with significant negative effects on the lymphoid lineage which leads to deficiencies in anti-infection activities, despite the beneficial effects on retarding hematopoietic stem cell (HSC) aging.^13^ Indeed other studies have also demonstrated considerable concerns on the severe immune-suppressive effects of DR in both humans and nonhumans.^14–18^ Therefore, for wider and more practical applications of DR, it would be important to explore the underlying mechanisms of how DR suppresses lymphoid system.

Gut microbiota plays important roles in shaping immunity.^19,20^ It has been shown that depletion of gut microbiota reduced dietary energy uptake and resulted in impaired murine hematopoiesis under undisturbed conditions as well as impaired immune reconstitution after bone marrow transplantation, which demonstrates links between gut microbiota, nutrition, and post-transplant hematopoiesis.^21,22^ Previously we have shown that DR reshapes gut microbiota in old mice to inhibit inflammation which is usually a result of activation of immune cells.^23^ Additionally, we have reported that DR remarkably attenuates inflammatory responses and improves survival rate of mice after exposure to lethal doses of chemotherapy, which is entirely dependent on the regulation of the DR gut microbiota.^24^ These studies indicate a potential link between regulation of gut microbiota and suppression of lymphopoiesis under DR condition, which remains to be explored.

As important role of gut microbiota is to ferment non-digestible carbohydrates from the diet to produce short chain fatty acids (SCFAs), which act as important molecular signals between the microbiota and host. A growing body of evidence suggests that SCFAs play a large multi-faceted role in the diet-gut microbiota-host axis.^25^ SCFAs, including formate, acetate, propionate and butyrate, are an important link between the microbiota and the immune system. Notably, it is shown that butyrate has a potential anti-inflammatory role which may involve inducing less responsiveness of immune cells to inflammatory signals.^25^ Glycolysis is known to serve as the major energy source for lymphopoiesis,^26–28^ which DR has been shown to inhibit and subsequently alleviate aging-related pathologies.^29^ It is unclear whether a potential link exists between the DR-induced diminishment of lymphopoiesis, DR-induced alteration in the gut microbiota, SCFA, and glycolysis in lymphoid cells.

In the current study, we show that the DR-induced diminishment of lymphopoiesis was significantly ameliorated by ablation of gut microbiota via administration of broad-spectrum antibiotics. Interestingly, glycolysis was significantly down-regulated in lymphoid cells of DR mice and inhibition of glycolysis reverted the rescue effect on lymphopoiesis by antibiotic-administration in DR mice. Furthermore, we show that DR remarkably reconstructed gut microbiota, with a significant increase in Lactobacillus and Bacteroides that are known butyrate-producing bacterial taxa. In line with this, expression of *But*, a butyrate synthesis gene encoding the key bacterial enzyme butyryl-CoA:acetate CoA-transferase (BCoAT), was elevated in the stool of DR mice. Moreover, extra butyrate feeding in AL mice down regulated glycolysis in lymphoid cells and mimicked the inhibition of lymphopoiesis in AL mice. Together, our study provides the first experimental evidence that gut microbiota mediates the inhibition on lymphopoiesis via down-regulation of glycolysis under DR condition, which is associated with increased synthesis of butyrate. Our study uncovered a candidate that could potentially be targeted to ameliorate the negative effects of DR on lymphopoiesis, and therefore may have important implications for the wider application of DR and promoting healthy aging.

## Results

### Gut microbiota mediates diminishment of lymphopoiesis under DR

**Figure 1.**
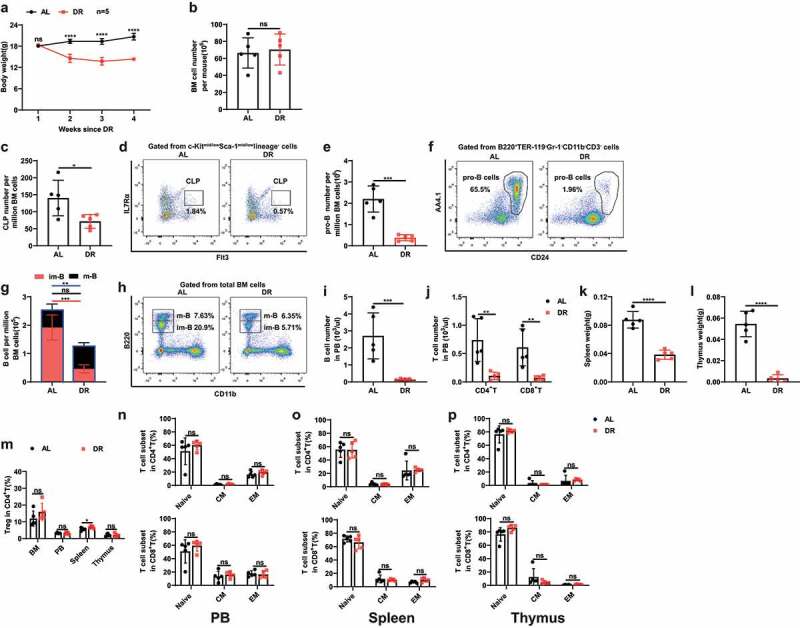
DR leads to significant diminishment in lymphoid lineages. 2–3 months old mice were fed with DR or AL diet for 4 weeks (n = 5 mice per group from 1 experiment representative of 2 independent experiments). (a) Body weight at indicated time points since DR. (b) Total number of BM cells of mice. (c-j) Frequencies of indicated populations determined by FACS. (k, l) Spleen and thymus weight. (m) Frequencies of Treg cells in BM, PB, spleen and thymus. (n-p) Frequencies of naïve T cells and memory T cells in PB (n), spleen (o), and thymus (p). Results were displayed as mean ± SD. ns, not significant; *p < .05; **p < .01; ***p < .001; ****p < .0001 by unpaired two-tailed Student’s t test. CM: Central memory T cells, EM: Effector memory T cells.

We first treated 2–3 months old mice with 30% DR for 4 weeks. DR resulted in a rapid body weight loss (Figure 1a). In line with our previous study,^13^ DR had no effect on the overall cell number in bone marrow but leads to significant loss of body weight and diminishment in lymphoid lineages, including common lymphoid progenitor cells (CLP = IL-7Rα^+^Flt3^+^c-Kit^mid/low^Sca-1^mid/low^lineage^−^ cells), pro-B cells (B220^+^CD24^+^AA4.1^+^TER-119^−^Gr-1^−^CD11b^−^CD3^−^ cells), B cells in bone marrow (in particular, B220^low^ immature B cells), B cells (B220^+^ cells) and T cells (CD4^+^ and CD8^+^ cells) in peripheral blood, accompanied by a notable shrinkage of spleen and thymus mass (Figure 1b-l). Further analysis on T cell subpopulations showed that DR increased regulatory T cells (Tregs: CD3^+^CD4^+^CD25^+^Foxp3^+^) in spleen but not in bone marrow (BM), peripheral blood (PB) and thymus (Figure 1m). DR showed neutral effects on naïve T cells and memory T cells, including central memory T cells (CM: CD4^+^CD44^+^CD62L^+^/CD8^+^CD44^+^CD62L^+^) cells and effector memory T cells (EM: CD4^+^CD44^+^CD62L^−^/CD8^+^CD44^+^CD62L^−^) in a homeostasis state (Figure 1n-p).

**Figure 2.**
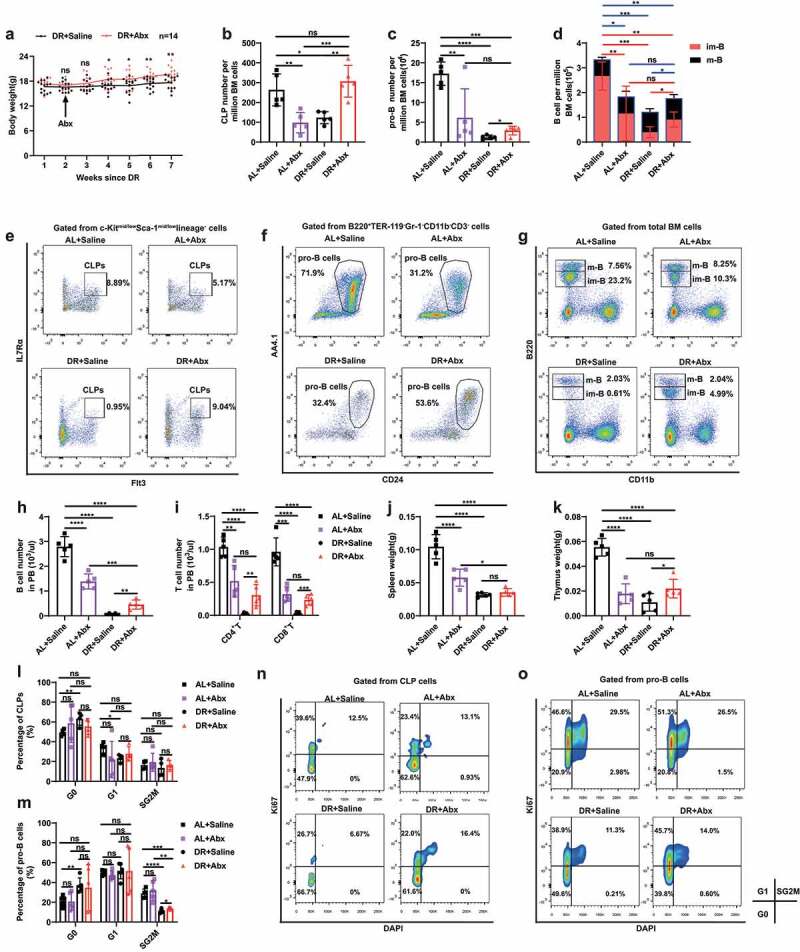
Gut microbiota mediates diminishment of lymphopoiesis under DR. 2–3 months old mice were put on DR or AL diet for 2 weeks, and afterward fed by gavage for 5 d and then in drinking water for 4 weeks with broad spectrum antibiotics or saline. (a) Body weight (n = 14 mice per group combined from 2 independent experiments). (b-i) Frequencies of indicated populations determined by FACS. (j, k) Spleen and thymus weight (n = 5 mice per group from 1 experiment representative of 2 independent experiments). (l-o) Quantification of CLPs and pro-B cells in indicated cell cycle phases (n = 4–5 mice per group from 1 experiment representative of 2 independent experiments) and representative FACS plots. Results were displayed as mean ± SD. ns, not significant; *p < .05; **p < .01, ***p < .001, ****p < .0001 by unpaired two-tailed Student’s t test.

To investigate the effect of gut microbiota on DR-induced suppression on lymphopoiesis, we applied broad-spectrum antibiotics (Abx) to AL and DR mice in order to ablate the gut microbiota. Intriguingly, wiping out the microbiota rescued the DR-induced body weight loss and significantly increased number of CLPs, pro-B cells, and B cells (in particular immature B cells) in bone marrow (Figure 2a-g). The numbers of B cells and T cells in peripheral blood were also elevated in Abx treated DR mice compared to saline treated DR mice (Figure 2h, i). Furthermore, Abx increased the weight of the thymus of DR treated mice, though the weight of spleen was not as significantly affected (Figure 2j, k). Previously, we have shown that DR suppresses proliferation of CLPs, which could contribute to the diminishment of lymphoid lineage in DR treated mice.^13^ To elucidate what mechanism could be involved in the rescue of lymphoid lineage, we further analyzed cell cycle activities of CLPs and pro-B cells in Abx treated DR mice. This analysis uncovered that Abx increased the number of pro-B cells in S/G2/M phase yet had neutral effect on the G0 and G1 cell cycles of CLPs (Figure 2l-o). On the contrary, Abx application in AL mice resulted in a significant reduction in numbers of CLPs, pro-B cells, and B cells in bone marrow, peripheral B cells and T cells, and in weight of spleen and thymus (Figure 2b-k), which was in line with a previous publication.^22^ Abx treatment did not change the cell cycle status of CLPs and pro-B cells in AL mice (Figure 2l-o). Comparing AL mice and DR mice, which both received Abx treatment, showed that the number of CLPs was significantly higher in DR-Abx mice, while the number of pro-B cells, B cells (including mature and immature B cells) in bone marrow, T cells in peripheral blood (including CD4^+^ and CD8^+^ cells), and weight of thymus were not significantly different between these two groups, indicating a remarkable rescue on lymphopoiesis by Abx application in DR mice (Figure 2b-g, i and k). Together, these data indicated that Abx treatment has distinct roles in lymphopoiesis of AL and DR mice, and that DR-shaped gut microbiota specifically contribute to the suppression of the lymphoid lineage, which may involve the increased proliferation of pro-B cells.

### Inhibition of glycolysis in lymphoid cells mediates the gut-microbiota alteration-induced suppression of lymphopoiesis under DR

**Figure 3.**
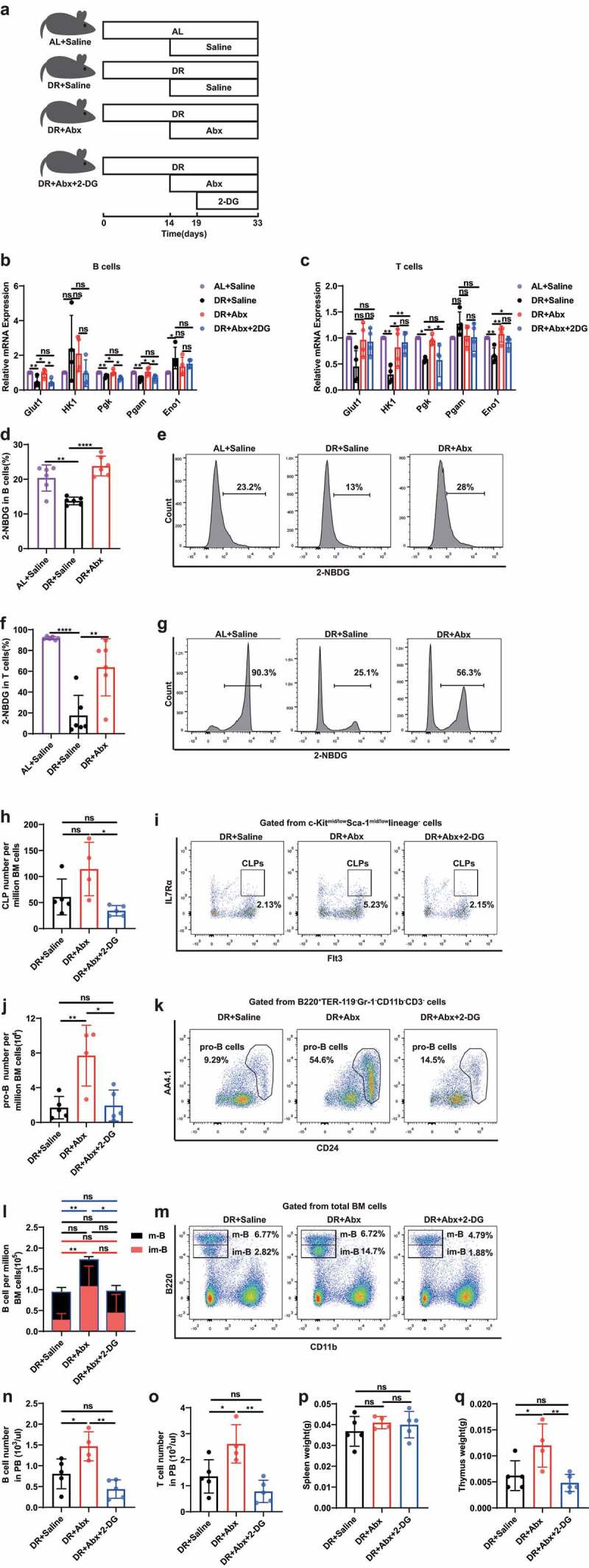
Inhibition of glycolysis in lymphoid cells mediates the gut-microbiota alteration-induced suppression of lymphopoiesis under DR. 2–3 months old mice were put on DR diet for 2 weeks, and afterward fed by gavage for 5 d and then in drinking water for 2 weeks with broad spectrum antibiotics or saline. Simultaneously, saline or 2-DG was administered by intraperitoneal injection once per day for 2 weeks. (a) Experimental scheme. (b and c) qPCR analysis of relative mRNA expression of indicated genes in sorted B220^+^ cells (b) or thymus cells (c). (d-g) 2-NBDG uptake by sorted B220^+^ cells (d, e) and thymus cells (f, g). Fluorescence derived from 2-NBDG was measured by flow cytometry (n = 6 mice per group from 1 experiment representative of 2 independent experiments). (d and f) Frequencies of 2-NBDG-positive cells in B220^+^ cells (d) and thymus cells (f). (e and g) Representative FACS plots of B220^+^ cells (e) and thymus cells (g). (h-o) Frequencies of indicated populations determined by FACS. (p and q) Weight of Spleen (p) and thymus (q) (n = 4–5 mice per group from 1 experiment representative of 2 independent experiments). Results were displayed as mean ± SD. ns, not significant; *p < .05; **p < .01; ****p < .0001 by One-way ANOVA test.

**Figure 4.**
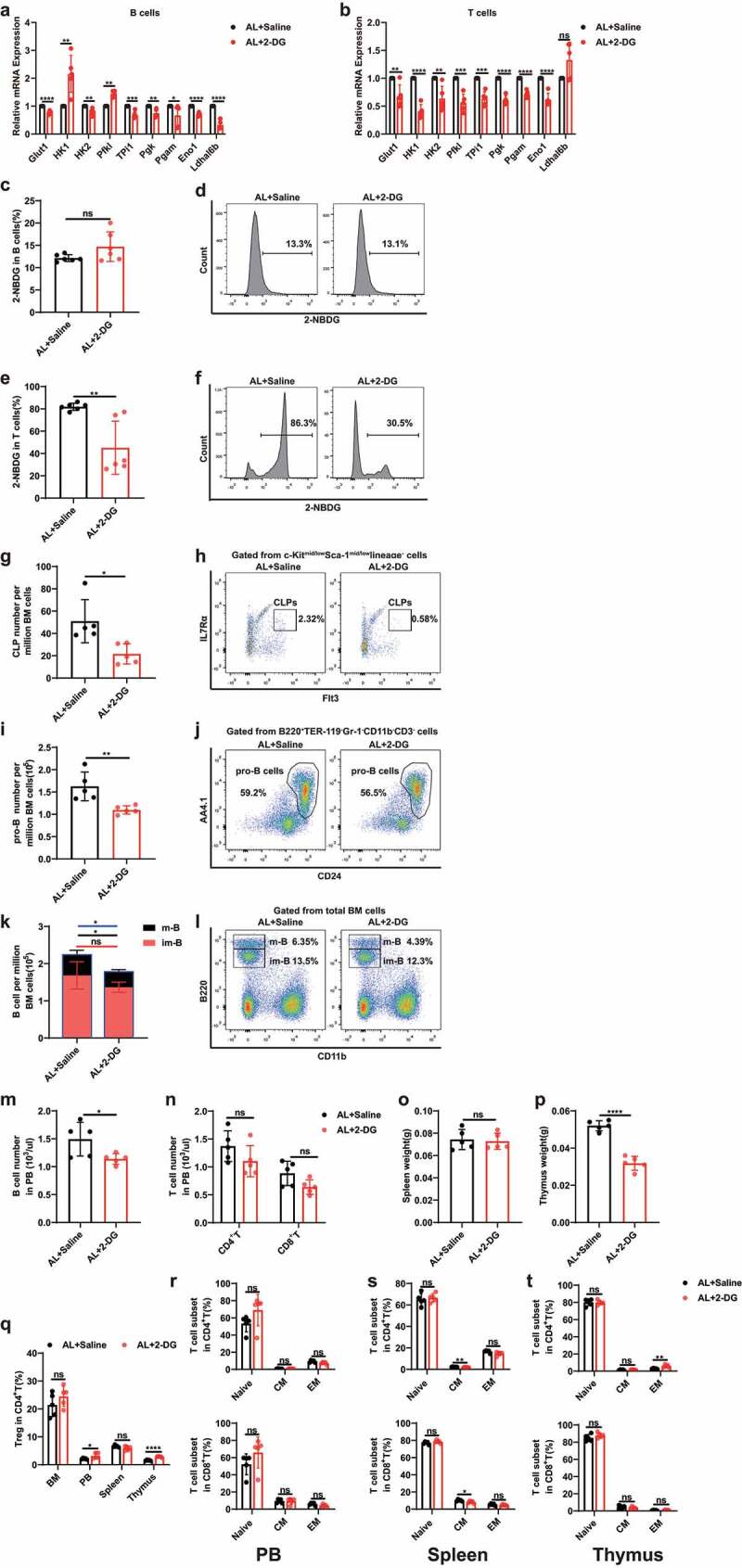
Inhibition of glycolysis in lymphoid cells leads to diminishment of lymphoid lineages in AL mice. (a and b) qPCR analysis of relative mRNA expression of indicated genes in sorted B220^+^ cells (a) or thymus cells (b). (c-f) 2-NBDG uptake by sorted B220^+^ cells (c, d) and thymus cells (e, f). Fluorescence derived from 2-NBDG was measured by flow cytometry (n = 6 mice per group from 1 experiment representative of 2 independent experiments). (c and e) Frequencies of 2-NBDG-positive cells in B220^+^ cells (c) and thymus cells (e). (d and f) Representative FACS plots of B220^+^ cells (d) and thymus cells (f). (g-n) Frequencies of indicated populations determined by FACS. (o, p) Spleen and thymus weight (n = 5 mice per group from 1 experiment representative of 2 independent experiments). (q) Frequencies of Treg cells in BM, PB, spleen and thymus. (r-t) Frequencies of naïve T cells and memory T cells in PB (r), spleen (s), and thymus (t) (n = 5 mice per group from 1 experiment representative of 2 independent experiments). Results were displayed as mean ± SD. ns, not significant; *p < .05; **p < .01; ***p < .001; ****p < .0001 by unpaired two-tailed Student’s t test.

It is known that proliferation of B and T lymphoid cells is largely dependent on glycolysis.^26–28,30^ We therefore analyzed sorted bone marrow B lymphocytes and thymus cells derived from mice under AL or DR conditions to examine the expression levels of key genes in the glycolysis pathway (Glut1, HK1, Pgk, Pgam, and Eno1). Interestingly, these genes were down regulated in B lymphocytes (Glut1, Pgk, and Pgam) and thymus cells (HK1, Pgk and Eno1) in DR mice, while their expression levels were restored when the gut microbiota was wiped out by Abx (Figure 3a-c). With pharmacological inhibition of glycolysis by 2-Deoxy-D-glucose (2-DG) administration in Abx-treated DR mice bringing down the expression levels of these genes to DR-saline levels. To directly measure the glucose uptake, a fluorescent glucose analog, 2-[N-(7-nitrobenz-2-oxa-1,3-diazol-4-yl) amino]-2-deoxy-glucose (2-NBDG) was used, allowing for direct quantification of glucose incorporation in living cells by flow cytometry.^31^ Compared to B lymphocytes and thymus cells derived from AL mice, the cells derived from DR mice showed a significant decrease in frequency of 2-NBDG-positive cells, while they were restored when the gut microbiota was wiped out by Abx, which indicates DR-shaped gut microbiota mediates a reduced glucose uptake in lymphocytes (Figure 3d-g). Along with the regulation of glycolytic gene expression and glucose uptake, Abx treatment rescued the diminishment of both B and T lymphoid lineages in DR mice. Additional inhibition of glycolysis by 2-DG administration in Abx-treated DR mice reverted the lymphopoiesis rescue effects seen in Abx treated mice (Figure 3h-q). To further study the inhibitory effect of glycolysis on lymphoid lineages, we administrated 2-DG in AL mice, which as expected led to down-regulation of genes in the glycolysis pathway in bone marrow B lymphocytes (including Glut1, HK2, TPi1, Pgk, Pgam, Eno1, and Ldhal6b) and thymus cells (including Glut1, HK1, HK2, Pfkl, TPi1, Pgk, Pgam, and Eno1) (Figure 4a, b). We observed that 2-DG significantly inhibited the glucose uptake in thymus cells, though had neutral effect on B lymphocytes by 2-NBDG assay (Figure 4c-f). Interestingly, 2-DG administration in AL mice also resulted in reduced number of lymphocyte progenitor cells and B cells in bone marrow and peripheral blood, and lower weight of thymus, though T cell number in peripheral blood and weight of spleen was not significantly altered (Figure 4g-p).

It has been shown that lowering glycolysis promoted T cell differentiation into regulatory T cells (Tregs) and increased the capacity of T cells to enter memory pool.^31,32^ We further investigated the effect of inhibition of glycolysis on the formation of T cell subsets such as Tregs and memory T cells after 2-DG administration, and we observed higher frequency of Tregs in the peripheral blood and thymus in 2-DG-treated mice (Figure 4q). However, 2-DG showed minimal effects on the frequency of memory T cells, including CM cells and EM cells in a homeostasis state (Figure 4r-t).

### DR significantly alters composition of the gut microbiota

**Figure 5.**
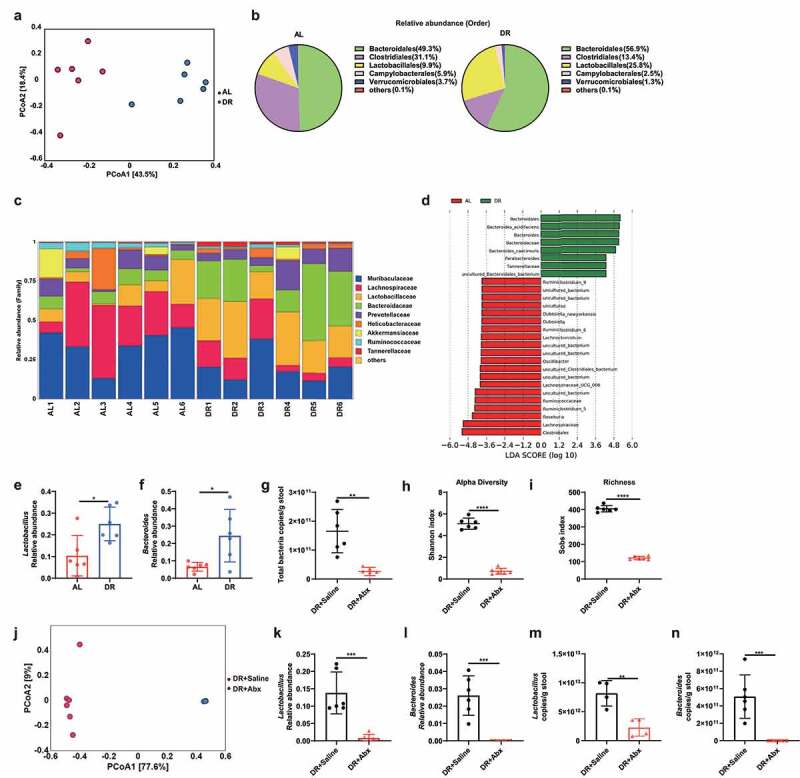
DR significantly alters composition of the gut microbiota. (a-f) 2–3 months old mice were fed with DR or AL diet for 4 weeks. Fecal samples were randomly collected from mice on the last day of the experiment for 16S rRNA gene sequencing (n = 6 mice per group from 1 experiment representative of 2 independent experiments). (a) Variation of intestinal flora structure of indicated groups along PC1 and PC2 of PCoA based on the Bray–Curtis distance. (b, c) Relative abundance of the intestinal flora of indicated groups on the Order level (b) and Family level (c) showed by 16S rRNA gene sequencing. (d) LDA scores in the fecal microbiomes of indicated groups. LDA score>2 were shown. (e, f) Relative abundance of the *Lactobacillus* (e) and *Bacteroides* (f) showed by 16S rRNA gene sequencing. (g-n) 2–3 months old mice were fed by gavage for 5 d after 2 weeks of DR diet, and then in drinking water for 4 weeks with broad spectrum antibiotics or saline. Fecal samples were randomly collected from mice on the last day of the experiment for qPCR analysis and 16S rRNA gene sequencing (n = 4–6 mice per group from 1 experiment representative of 2 independent experiments). qPCR analysis of the amounts of total bacteria (g), Shannon index (h) and sobs index (i) of indicated groups based on alpha diversity analysis, variation of intestinal flora structure of indicated groups along PC1 and PC2 of PCoA based on the Bray–Curtis distance(j). (k, l) Relative abundance of the *Lactobacillus* (k) and *Bacteroides* (l) showed by 16S rRNA gene sequencing. (m, n) qPCR analysis of the amounts of *Lactobacillus* (m) and *Bacteroides* (n) from fecal DNA. Results were displayed as mean ± SD. ns, not significant; *p < .05; **p < .01; ***p < .001; ****p < .0001 by unpaired two-tailed Student’s t test.

To begin to understand the underpinning of these effects, we first analyzed the composition of gut microbiota of mice treated with 30% DR or AL for 4 weeks by 16S rRNA gene deep-sequencing (Illumina 250 bp paired-end). Interestingly, the overall structure of the gut microbiota was significantly shifted in DR treated mice as shown by principal-coordinate analysis (PCoA) based on Bray-Curtis distance (Figure 5a), with remarkable increases of Bacteroidales, Lactobacillales, and a decrease of Clostridiales (Figure 5b, c). A linear discriminant analysis (LDA) score over 2 was used for further investigation of top regulated bacteria taxa. The analysis showed that the Order of Bacteroidales, Family of Bacteroidaceae and Genus of Bacteroides were among the top five enriched taxa in the intestines of DR mice (Figure 5d). In particular, the relative abundance of Lactobacillus and Bacteroides was significantly increased in DR mice (Figure 5e, f).

As anticipated, Abx application led to massive ablation of the total amount of gut microbiota and the bacterial taxa diversity in DR mice as shown by lower copies of total bacteria in the stool, reduced Shannon index and Sobs index, and PCoA (Figure 5g-j). Coupled with that, qPCR analysis showed a remarkable diminishment in the copy number of Lactobacillus and Bacteroides upon antibiotic treatment (Figure 5k-n). Together, the results indicated that bacterial richness and the amount of Lactobacillus and Bacteroides in DR mice were severely compromised by antibiotic treatment (Figure 5g-n). Since Abx application significantly rescued lymphoid diminishment under DR condition (Figure 2), it supports the scenario that DR-induced alteration of the gut microbiota mediates its inhibitory effect on lymphopoiesis.

### Supplementary butyrate in AL mice mimics the impact of DR on lymphopoiesis

To dissect the role of DR-induced alteration of gut microbiota in the diminishment of lymphopoiesis, we first sought to mimic the effect by gastric gavage of *Lactobacillus rhamnosus* GG (LGG). LGG was chosen because a higher relative abundance of *Lactobacillus* genus was observed in DR mice and LGG belonging to the Lactobacillales lactic acid bacteria Order. We applied LGG to AL mice for 5 weeks and observed a significant loss of body weight (Supplementary Fig. 1A). LGG gavage led to decreased number of pro-B cells and immature B cells, as well as pro-B cells in resting phase, however showed neutral effects on CLPs, total B cells in bone marrow, B and T lymphocyte in peripheral blood, spleen and thymus (Supplementary Fig. 1B-L). These results indicate that LGG only partially mimics the inhibition effect of DR on lymphoid lineage.

**Figure 6.**
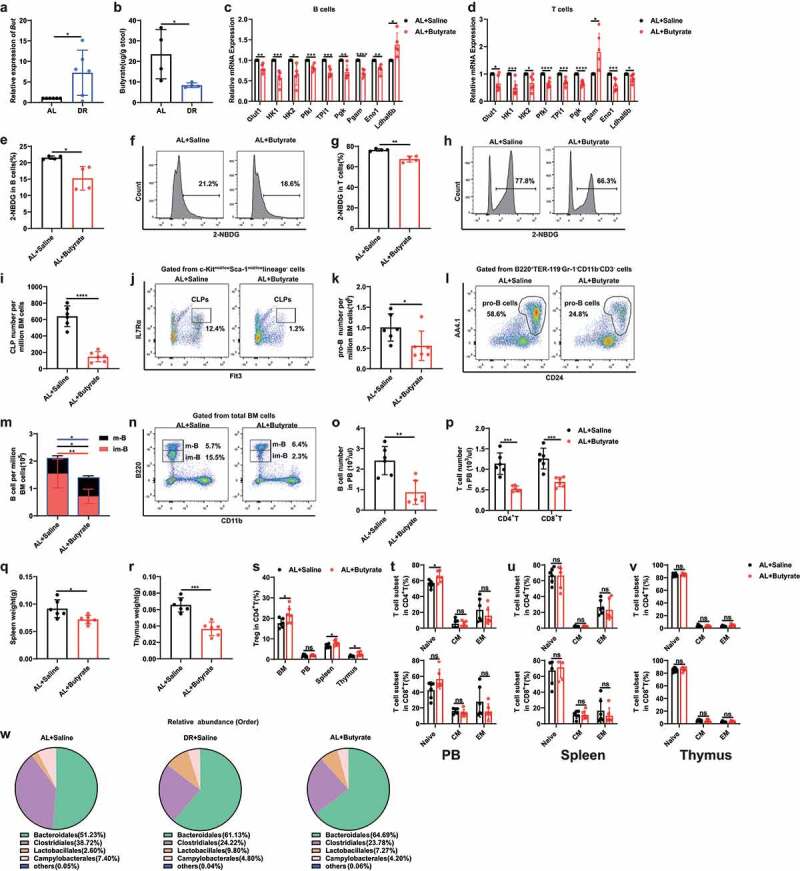
Supplementary Butyrate in AL mice mimics the impact of DR on lymphopoiesis. (a, b) 2–3 months old mice were fed with DR or AL diet for 4 weeks. Fecal samples were randomly collected from mice on the last day of the experiment. (a) Relative expression of *But* by qRT-PCR analysis (n = 6 mice per group from 1 experiment representative of 2 independent experiments). (b) Butyrate concentration analysis by GC-MS (n = 4 mice per group from 1 experiment representative of 2 independent experiments). (c-v) 2–3 months old mice were orally administered with butyrate (6 M, Sigma-Aldrich) dissolved in saline for 8 d or saline as a control. (c and d) qPCR analysis of relative mRNA expression of indicated genes in sorted B220^+^ cells (c) and thymus cells (d) (n = 5 mice per group from 1 experiment representative of 2 independent experiments). (e-h) 2-NBDG uptake by B220^+^ cells (e, f) and thymus cells (g, h). Fluorescence derived from 2-NBDG was measured by flow cytometry (n = 4 mice per group from 1 experiment representative of 2 independent experiments). (e and g) Frequencies of 2-NBDG-positive cells in B220^+^ cells (e) and thymus cells (g). (f and h) Representative FACS plots of B220^+^ cells (f) and thymus cells (h). (i-p) Frequencies of indicated populations determined by FACS. (q and r) Weight of spleen (q) and thymus (r) (n = 6 mice per group from 1 experiment representative of 2 independent experiments). (s) Frequencies of Treg cells in BM, PB, spleen and thymus. (t-v) Frequencies of naïve T cells and memory T cells in PB (t), spleen (u), and thymus (v) (n = 6 mice per group from 2 independent experiments). (w) Fecal samples were randomly collected from mice on the last day of the experiment for 16S rRNA gene sequencing. Relative abundance of the intestinal flora of indicated groups on the Order level (n = 5 mice per group from 1 experiment representative of 2 independent experiments). Results were displayed as mean ± SD. ns, not significant; *p < .05; **p < .01; ***p < .001; ****p < .0001 by unpaired two-tailed Student’s t test.

Gut microbiota ferment non-digestible carbohydrates from diet to produce short chain fatty acids (SCFA) which act as important molecular signals between microbiota and host, with the SCFA butyrate thought to have potential roles in glucose homeostasis and inhibition of inflammatory responses.^25,33^ Since the most increased bacteria taxa Lactobacillus and Bacteroides in DR mice are known to produce butyrate, we asked whether butyrate production was promoted under the DR condition. A core gene for bacterial butyrate production is *But* (butyryl-CoA:acetate-CoA transferase) which encodes butyryl-CoA:acetate-CoA transferase. Indeed, quantitative PCR proved an increased expression of *But* in the stool of DR mice (Figure 6a), though the fecal level of butyrate itself was reduced in DR mice comparing to AL mice (Figure 6b). It has been shown that 95% of colonic SCFAs are absorbed with 5% excreted in feces.^34^ Therefore, we speculated that this could have resulted from up-regulated utilization of butyrate by colonocytes under DR condition. To further decipher potential roles of butyrate in mediating the inhibition of glycolysis and lymphopoiesis, we fed AL mice with butyrate following a previously published protocol.^35^ Interestingly, expression levels of several genes in the glycolysis pathway, were decreased in bone marrow B lymphocytes (including Glut1, HK1, HK2, Pfkl, Tpi1, Pgk, Pgam and Eno1) and thymus cells (including Glut1, HK1, HK2, Pfkl, Tpi1, Pgk, Eno1 and Ldhal6b) of butyrate-fed mice (Figure 6c, d). In line with that, 2-NBDG assay also showed a significantly reduced frequency of 2-NBDG-positive cells in B lymphocytes and thymus cells of butyrate-fed mice, indicating lower glucose uptake in these cells in mice after butyrate administration (Figure 6e-h). Furthermore, administration of butyrate led to lower number of CLPs, pro-B cells, B and T lymphocyte in bone marrow and peripheral blood, and reduced weight of spleen and thymus (Figure 6i-r). We further investigated the effect of butyrate administration on T cell subpopulations, such as Tregs and memory T cells, which were induced in culture upon IL-2 stimulation after butyrate treatment.^36,37^ Consistent with that, we observed a higher frequency of Tregs in BM, spleen, and thymus in butyrate-fed mice (Figure 6s). However, the frequency of memory T cells, including CM cells and EM cells, was not altered by butyrate administration in vivo in a homeostasis state (Figure 6t-v). Intriguingly, the gut microbiota was re-shaped toward a more “DR-like” mode in butyrate-fed mice with elevated proportion of Bacteroidales (64.69%) and Lactobacillales (7.27%) when compared to saline-fed mice (Bacteroidales (51.23%) and Lactobacillales (2.60%) (Figure 6w)).

### Butyrate inhibits proliferation and survival of human lymphoma cells in culture

To further investigate the inhibitory effect of butyrate in a human context, we cultured a diffuse large B cell lymphoma human cell line (SUDHL-4) and applied butyrate at different concentrations in the culture system. Interestingly, though 0.25 mmol/L butyrate showed neutral effects on the cell growth and survival, 0.5 mmol/L butyrate significantly reduced cell growth with decreased cell number and the fewer cells in G2/M phase and a small rise in apoptosis as indicated by Caspase-1 positive cells detected by Flowcytometry analysis (Supplementary Fig. 2A-C). Butyrate at higher concentration (1 mmol/L) induced further inhibition on cell proliferation and a significant induction of apoptosis (Supplementary Fig. 2A-C). These results indicated that butyrate also had inhibition effects on cell growth and survival on human lymphoid cells.

## Discussion

For a long time DR has been promising hope to reduce the burden of aging societies as it promotes healthy aging. However, we and others have uncovered significant inhibitory effects caused by DR on the immune system that could reduce the overall benefits one could get from therapeutic DR. It is therefore important to explore the underlying mechanisms that could mediate the inhibition of lymphopoiesis by DR. The current study provides the first experimental evidence that DR restructures the gut microbiota, which in turn mediates a down-regulation of glycolysis in lymphoid lineages with subsequent inhibition of B and T lymphopoiesis. In addition, we further reveal that DR led to an increase of butyrate-producing bacteria in the gut, with supplemental butyrate-feeding in AL mice able to mimic the restructuring of the gut microbiota and diminishment in lymphopoiesis observed in the DR condition. The current study provides an important clue that could be further explored to minimize the negative effects of DR on lymphopoiesis. A limitation of this study was that only female mice were used in all experiments, which should introduce certain consideration of the generalizability of the results due to the potential variability between different genders.

Gut microbiota are the first cells to sense the availability of host intestinal nutrients and directly participate in the catabolism, absorption and utilization of ingested food. Therefore, the gut microbiota and their metabolic products would change drastically under DR and could subsequently mediate some of the effects of DR on their hosts. Our previous study has shown that DR can induce a structural change in the gut microbiota that provides a local protection to the intestinal epithelial cells (including the intestinal stem cells) via a significant inhibition of inflammatory responses upon high dose-methotrexate treatment.^24^ The current study discloses a systemic effect on lymphopoiesis inhibition by DR-induced change in gut microbiota which down-regulates glycolysis. To further explore a potential factor that could mediate the change in gut microbiota to the systemic effect in the host, the study examined the effect of butyrate, a major SCFA which are products of bacterial fermentation of dietary fiber.^38^ Acetate, propionate and butyrate typically correspond to 90–95% of total SCFA in a 3:1:1 ratio.^39^ Butyrate has been shown to be a major energy source for colonocytes and could prevent the progression of colitis.^40,41^ The current study found that the most increased bacteria taxa in DR mice were Lactobacillus and Bacteroides which are known to produce butyrate. Moreover, the expression of *But*, a core gene for bacterial butyrate production, was found to be elevated in DR mice, and supplemental feeding of butyrate to AL mice could mimic the structural changes seen in the gut microbiota of DR mice, and likewise inhibit glycolysis and lymphopoiesis. It is tempting to speculate that DR restructures the gut microbiota with more butyrate-producing bacteria taxa which promotes butyrate utilization by the colonic epithelium to compensate for the caloric energy reduction present under DR, leading to down-regulated glycolysis in lymphoid lineages and diminishment of the lymphoid lineage. How local butyrate changes in the intestine could result in systematic alteration in lymphopoiesis remains to be elucidated. Whether this change is caused by butyrate *per se*, metabolic signaling or alternative mechanism is unclear. Nevertheless, this study proposes a scenario of how dietary restriction could systemically impact on immune system via regulation on the gut microbiota, though the evidence presented here is only associative.

Our studies have indicated that DR mainly increased Lactobacillales and Bacteroidetes in the intestine. The current study showed that extra feeding of butyrate phenocopied the inhibition of lymphopoiesis by DR, while LGG feeding only minimally mimicked the phenotype. Further research is needed to disentangle which bacterial species contribute most to butyrate synthesis in the intestine under DR conditions as well as the involvement of other SCFAs.

Of note, spleen was not as significantly affected as the thymus by intervention on gut microbiota in the current study. It is known that thymus is a central immune organ where CLPs develop into downstream T cells which will further differentiate and mature in the thymus.^42,43^ Spleen, on the other hand, is a peripheral immune organ which accommodates mature lymphocytes.^44,45^ These results may indicate that progenitor and immature lymphoid cells were more affected by the gut microbiota and glycolysis under the DR condition. We further investigated the effects of lower glycolysis (including DR, 2-DG treatment, and butyrate treatment) on Tregs and memory T cells. In general, we observed an increased frequency of Tregs under low glycolysis conditions which were consistent with previously published data.^32,46^ However, the frequency of memory T cells was not altered by DR or butyrate administration and was only minimally changed by 2-DG administration in vivo in a homeostasis state. Previously published data showed that inhibition of glycolysis by 2-DG administration enhanced the generation of memory cells from activated CD8^+^ T cells upon cognate gp100 peptide infection.^31^ It will be interesting to investigate differentiation of T cells in DR mice in an infection model in the future. Furthermore, previously we have reported that DR improves HSC functionality during aging despite its negative role on lymphopoiesis. The current study suggested that the restructured gut microbiota only impacted on lymphopoiesis with HSCs unaffected, indicating that the effects of DR on hematopoietic system involve different mechanisms.

Together, the current study proposes a model of how dietary restriction regulates the gut microbiota and its products, which play an important role in the metabolism of lymphoid cells and their generation. Therefore, the current study uncovered an important and novel mechanism of how DR elicits its negative effect on the immune system, which could be further explored for ameliorating it and promoting healthy aging.

## Materials and methods

### Mice and dietary restriction

C57BL/6 J female mice were purchased from Hunan SJA Laboratory Animal Co. Ltd. and maintained in pathogen-free conditions on a 12-h light/dark cycle at 23°C–25°C at the animal facilities of Nanchang Royo Biotech. DR was performed as previously described.^47^ DR mice were housed individually and were given daily food – 70% of the normal amount of food consumed by body weight- and gender-matched ad libitum (AL) mice at timed intervals. For the AL group, mice were fed with an unlimited amount of food. All mouse experiments were approved by the Animal Experimental Ethical Inspection of Nanchang Royo Biotech Co. Ltd (RYE2019030501).

### Flow cytometry

Flow cytometry was performed as previously described.^47^ Mouse BM cells were obtained by crushing all hind limbs and pelvis in sterile PBS supplemented with 2% heat-inactivated fetal bovine serum (FBS) and filtered with 40-μm cell strainer. Afterward, the BM cells were resuspended in red cell lysis buffer to remove the red cell. Then, BM cells were washed with PBS, counted by the cell counter, and were incubated with antibody cocktails for flow cytometry analysis to detect phenotypic cell surface markers as follows. Common lymphoid progenitors (CLPs, IL-7Rα^+^Flt3^+^c-Kit^mid/low^Sca-1 ^mid/low^lineage^−^ cells) were detected using a lineage cocktail (biotinylated anti-TER-119, -Gr-1, -B220, -CD11b, -CD3, -CD4, and -CD8 antibodies; BioLegend) and APC anti-mouse c-Kit (2B8; BioLegend), PE-Cy7 anti-mouse Sca-1 (D7; BD), PerCP-Cy5.5 anti-mouse IL-7Rα (A7R34; eBioscience), PE anti-mouse Flt3 (A2F10; BioLegend) and APC-Cy7 Streptavidin (BioLegend). Progenitor B cells (pro-B cells, B220^+^CD24^+^AA4.1^+^TER-119^−^Gr-1^−^CD11b^−^CD3^−^ cells) were detected using a lineage cocktail (biotinylated anti-Gr-1, -CD11b, -TER- 119, and -CD3 antibodies) and PerCP-Cy5.5 anti-mouse CD19 (1D3/CD19; BioLegend), PE-Cy7 anti-mouse B220 (RA3-6B2; BioLegend), PE anti- mouse CD43 (S7; BD), APC anti-mouse AA4.1 (AA4.1; BioLegend), FITC anti-mouse CD24 (M1/69; BioLegend) and APC-Cy7 Streptavidin (BioLegend). Differentiated BM

cells were detected using PE-Cy7 anti- mouse B220 (RA3-6B2; BioLegend), APC-Cy7 anti-mouse CD11b (M1/70; BioLegend), APC anti-mouse CD4 (RM4-5; BioLegend), and PerCP-Cy5.5 anti-mouse CD8 (53–6.7; BioLegend) antibodies. For regulatory T cells (Tregs: CD3^+^CD4^+^CD25^+^Foxp3^+^) and Naïve/ memory T cells (central memory T cells: CD4^+^CD44^+^CD62L^+^/CD8^+^CD44^+^CD62L^+^; effector memory T cells: CD4^+^CD44^+^CD62L^−^/CD8^+^CD44^+^CD62 L^−^) analysis, FITC anti-mouse CD3 (145–2C11; BioLegend), APC anti-mouse CD4 (RM4-5; BioLegend), PerCP-Cy5.5 anti-mouse CD8 (53–6.7; BioLegend), PE-Cy7 anti-mouse CD25 (PC61; BioLegend), APC-Cy7 anti-mouse CD44 (IM7; BioLegend), BV421 anti-mouse CD62 L (MEL-14; BioLegend) were used for cell surface staining. Afterward, the cells were fixed and permeabilized with the Transcription Factor Buffer Set (BD) according to the manufacturer’s instructions and subjected to intracellular Foxp3 staining using PE anti-mouse Foxp3 (150D; BioLegend). After staining, cells were analyzed on a flow cytometer (FACS Canto II; BD).

For cell cycle analysis, BM cells were stained with surface markers as described in the previous paragraph, and fixed and permeabilized with the Cytofix/Cytoperm Fixation/Permeabilization Solution kit (BD) according to the manufacturer’s instructions. Afterward, cells were stained with FITC anti-mouse Ki67 (B56; BD) for 1 h on ice and incubated with DAPI/PBS medium to stain for DNA content. For human lymphoma cell line cell cycle analysis, the cells were processed using the Cell Cycle Detection Kit (KeyGEN BioTECH) according to the manufacturer’s instructions. Afterward, the cells were subjected to cell cycle analysis by flow cytometry.

For active caspase 1 staining, the cells were incubated with FAM-YVAD-FMK fluorescent probe (FAM-FLICA® Caspase-1 Assay Kit, Immunochemistry Technologies) to label active caspase-1 enzyme in living cells according to the manufacturer’s instructions and analyzed using flow cytometry.

For 2-NBDG uptake assay, sorted B220^+^ cells and thymus cells were incubated with sugar-free RPMI-1640 medium with 100 μM fluorescent glucose analog 2-NBDG (Absin, China) for 30 minutes in an incubator. Then, the medium was washed off with PBS, and the fluorescence of the samples was monitored at an excitation wavelength of 465 nm and an emission wavelength of 540 nm.

### Sorting

For B220^+^ cells sorting, BM cells were labeled with APC

anti–mouse B220 antibody, and B220^+^ cells were enriched and sorted using anti-APC magnetic beads and LS columns following the manufacturer’s instructions (Miltenyi Biotec).

### Peripheral blood cell counting

Peripheral blood (PB) was collected from the orbital venous plexus into tubes containing 0.5 M EDTA. PB cell count was assessed by an automated hematology analyzer (Sysmex, XS-500i).

### Antibiotic and 2-DG treatment

Antibiotics (Abx) treatment was performed as previously described.^24^ Mice received broad-spectrum antibiotics (ampicillin, neomycin, metronidazole and vancomycin (10 mg/mouse/d)) via gastric gavage (0.2 ml) for 5 d followed by administration in drinking water (ampicillin, neomycin and metronidazole: 1 g/L; vancomycin: 500 mg/L) until the end of the experiment. For the control group, saline was administrated in the same way as the antibiotics.

2-DG (2-deoxy-D-glucose, MedChemExpress) was dissolved in saline and administered by 1 g/kg intraperitoneal injection as a single dose daily. For the control group, saline was administrated in the same way as the 2-DG.

### LGG and butyrate treatment

LGG administration was performed as previously described.^24^ Mice were orally inoculated with LGG (ATCC 53103) (Bayer) (5 × 10^9^ CFU per 200 μl of saline) daily for 7 d and then orally once every 3 d until the end of the experiment according to the manufacturer’s instructions.

For butyrate administration, butyrate (Sigma-Aldrich) was dissolved in saline and diluted in drinking water at a final concentration of 6 mol/L for gavage. Drinking water containing same concentration of saline was prepared as control solution. Butyrate group and control group were administered continuously by daily gavage of 150 μl of prepared butyrate solution or control solution for 8 d.

### Butyrate measurements

Fecal butyrate concentrations were measured by gas chromatography-mass spectrometry. Briefly, cecal contents (150 mg) were homogenized in 2.5 mL of dichloromethane using a vortex mixer for 30 min and centrifuged at 8000 rpm for 5 min at 4°C, and the supernatant was collected. The extraction was repeated once. The volume was diluted to 5 ml with dichloromethane, with 0.9 ml taken and added to 0.1 ml BSTFA, derivatized at 60°C for 50 min, and finally passed through a 0.22 µm filter membrane for detection by GC-MS (Agilent 7890B-5977) with the following parameters: injector volume, 2 μL; split ratio, 10:1; injector temperature, 250°C; and carrier gas, helium.

### Fecal sample collection

Fresh fecal samples were collected in microtubes on ice and stored at −80°C within 1 h until DNA isolation for 16S rRNA gene sequencing.

### Fecal DNA isolation

Fecal samples were weighed and total DNA was extracted according to the operating instructions of Fecal Genome Extraction Kit (DP328; Tiangen Biotech). DNA concentration and purity were measured with Nanodrop 2000 (Thermo scientific).

### RNA isolation and cDNA synthesis

Total RNA was isolated from freshly sorted cells by using RNAsimple Total RNA Kit (TianGen Biotech) and reverse transcriptions were performed to synthesize first strand DNA by using RevertAid First Strand cDNA Synthesis kit (Thermo scientific) according to the manufacturer’s instructions with a procedure of incubation at 42°C for 60 min and heating inactivation at 70°C for 5 min.

### Quantitative Real-Time PCR (qPCR)

For sorted cells, qRT-PCR was performed using TransStart Tip Green qPCR SuperMix (TransGen Biotech) with an ABI 7900 Real-Time PCR system (Applied Biosystems) in triplicates. Relative expression of genes was normalized to β-actin in each sample and was normalized to 1 in the AL group. Primer sets are listed in Table S1.

For fecal samples, qPCR assays were performed using Ace qPCR SYBR Green Master Mix kit (Vazyme) with Bio-RAD 9600A system, as previously described.^24^ Primer sets are listed in Table S2.

### Microbial DNA extraction, PCR amplification and 16S rRNA gene sequencing

According to a previous publication,^24^ microbial DNA was extracted from fecal sample using DNA extraction kit (Minkagene Stool DNA kit) and 16S rRNA gene regions V3-V4 were amplified using universal primers (338 F 5ʹ-ACTCCTACGGGAGGCAGCAG-3ʹ and 806 R 5ʹ-GGACTACCAGGGTATCTAAT-3ʹ) with 12 bp barcode and TaKaRa Premix Taq® Version 2.0.

The length and concentration of the PCR product were detected by 1% agarose gel electrophoresis. PCR products were mixed in equimolar ratios according to the GeneTools Analysis Software (Version4.03.05.0, SynGene). Then, the PCR mixture was purified with EZNA Gel Extraction Kit (Omega, USA). Lastly, sequencing libraries were generated using NEBNext® Ultra™ DNA Library Prep Kit for Illumina® (New England Biolabs, USA) and sequenced on an Illumina Hiseq 2500 platform.

### Sequencing data processing

Quality filtering on the paired-end raw reads was performed under specific filtering conditions to obtain the high-quality clean reads according to the fastp (an ultra-fast all-in-one FASTQ preprocessor, V0.14.1, https://github.com/OpenGene/fastp) quality controlled process. Paired-end clean reads were merged using usearch software (V10, http://www.drive5.com/usearch/) according to the relationship of the overlap between the paired-end reads and the spliced sequences were called Raw Tags. Sequences were assigned to each sample based on their unique barcode and primer using cutadapt software (https://github.com/marcelm/cutadapt/), after which the barcodes and primers were removed and got the effective Clean Tags.

### OTU cluster and species annotation

Usearch software (V10, http://www.drive5.com/usearch/) was used for sequence analysis. Sequences with ≥97% similarity were assigned to the same OTU, which is assumed to represent a species. The sequence that occurred most frequently was used as a representative sequence for each OTU, which was then screened for further annotation.

### Human lymphoma cell line cuture

Human diffused large B cell lymphoma cell line SUDHL-4 was purchased from the Cell Bank of the Chinese Academy of Sciences (Shanghai, China). The cells were cultured at 37°C in 5% CO2 and 100% humidity in RPMI-1640 medium supplemented with 10% fetal bovine serum (Gibco) in 75 cm^2^flasks. Butyrate was applied at concentrations of 0.25, 0.5, and 1 mmol/L in the culturing system.

### Statistical analysis

GraphPad Prism 7.0 software was used for statistical analysis. The unpaired two-tailed Student’s t-test was used for two-group datasets, and one-way ANOVA was used for multi-group datasets to calculate p-values.

## Supplementary Material

Supplemental Material

## Data Availability

Raw illumina sequence data of the 16SrRNA gene generated in the study are available in the NCBI Sequence Read Archive (SRA) under accession number PRJNA781021 https://www.ncbi.nlm.nih.gov/bioproject/PRJNA781021/.
