# Supplementary material for: Gut microbiota mediates the inhibition of lymphopoiesis in dietary-restricted mice by suppressing glycolysis
Source: Gut Microbes. 2022 Sep 1;14(1):2117509. doi: 10.1080/19490976.2022.2117509 (PMC9450896; doi:10.1080/19490976.2022.2117509)
Supplement: Supplemental Material [file KGMI_A_2117509_SM3008.zip › Tao and Wang et al_Supplementary Figure legend.docx]

**Supplementary figure 1. LGG gavage** **partially** **mimics the inhibition effect of DR on lymphoid lineage.** 2-3 months old mice were orally inoculated with LGG or vehicle control for 5 weeks (n = 5 mice per group from 1 experiment representative of 2 independent experiments). (A) Body weight. (B-F, I, J) Frequencies of indicated populations determined by FACS. (G, H) Quantification of pro-B cells in indicated cell cycle phases and representative FACS plots. (K and L) Weight of spleen (K) and thymus (L). Results were displayed as mean ± SD. ns, not significant; *p < 0.05; **p < 0.01 by unpaired two-tailed Student's t test.

**Supplementary figure 2. Butyrate inhibits proliferation and survival of human lymphoma cells in culture.** SUDHL-4 cells were cultured at indicated concentrations and harvested for cell counting and cell cycle analysis or apoptosis on day 2 of culture (n =3-6 per group from 1 experiment representative of 2 independent experiments). (A) Cell counting. (B) Cell cycle analysis and representative plots by FACS. (C) Apoptosis analysis by Caspase-1 FACS and representative plots.
