# Supplementary material for: Gut microbiota mediates the inhibition of lymphopoiesis in dietary-restricted mice by suppressing glycolysis
Source: Gut Microbes. 2022 Sep 1;14(1):2117509. doi: 10.1080/19490976.2022.2117509 (PMC9450896; doi:10.1080/19490976.2022.2117509)
Supplement: Supplemental Material [file KGMI_A_2117509_SM3008.zip › Tao and Wang et al_Table S1.docx]

**Table S1. Primer list of glycolysis genes**

| **Genes** | **Forward Sequence** | **Reverse Sequence** |
| --- | --- | --- |
| Glut1 | 5'-AAACATGGAACCACCGCTAC-3' | 5'-AGGCCAACAGGTTCATCATC-3' |
| Hk1 | 5'-GAGGGATGCTGTAAAAAGGAGA-3' | 5'-TTCATAAGCACAAGTCATCATGG-3' |
| Hk2 | 5'-GCTGAAGGAAGCCATTCG-3' | 5'-TCCCAACTGTGTCATTTACCAC-3' |
| Pfkl | 5'-TCTCATCCAGCTACGTGAAGG-3' | 5'-CCTCCTCGCTGTACATGACC-3' |
| Tpi1 | 5'-ACCGAGAAGGTCGTGTTCGA-3' | 5'-GGCCAGGACCACCTTGCT-3' |
| Pgk | 5'-CCAAGGCTTTGGAGAGTCC-3' | 5'-GATCAGCTGGATCTTGTCTGC-3' |
| Pgam | 5'-CCTCATGGTGATTTTTAACCCTAA-3' | 5'-AAGATTGATCCCAACCTTCTAGG-3' |
| Eno1 | 5'-GGGTGATGAGGGTGGATTC-3' | 5'-ATCCATGCCAATGACAACCT-3' |
| Ldhal6b | 5'-CCAAGTGTGGGGTGAAGC-3' | 5'-TGGATGCTACACAACAGGAGA-3' |
| β-actin | CTAAGGCCAACCGTGAAAAG | ACCAGAGGCATACAGGGACA |
