# Supplementary material for: Gut microbiota mediates the inhibition of lymphopoiesis in dietary-restricted mice by suppressing glycolysis
Source: Gut Microbes. 2022 Sep 1;14(1):2117509. doi: 10.1080/19490976.2022.2117509 (PMC9450896; doi:10.1080/19490976.2022.2117509)
Supplement: Supplemental Material [file KGMI_A_2117509_SM3008.zip › Tao and Wang et al_Table S2.docx]

**Table S2. Primer list of Bacteria and *But***

| **Gene** | **Primer** | **Sequence** |
| --- | --- | --- |
| V4–V5 region | 515F | GTGCCAGCMGCCGCGGTAA |
|  | 907R | CCGTCAATTCMTTTRAGTTT |
| *But* | F | GCIGAICATTTCACITGGAAYWSITGGCAYATG |
|  | R | CCTGCCTTTGCAATRTCIACRAANGC |
| *Lactobacillus* | F | TGGAAACAGRTGCTAATACCG |
|  | R | GTCCATTGTGGAAGATTCCC |
| *Bacteroides* | F | CTGAACCAGCCAAGTAGCG |
|  | R | CGCCCGCCGCGCGCGGCGGGCGGGGCGGGGGCACGGGGGGCCGCAAACTTTCACAACTGACTTA |
