# Supplementary figures and images for: Gut microbiota mediates the inhibition of lymphopoiesis in dietary-restricted mice by suppressing glycolysis
Source: Gut Microbes. 2022 Sep 1;14(1):2117509. doi: 10.1080/19490976.2022.2117509 (PMC9450896; doi:10.1080/19490976.2022.2117509)

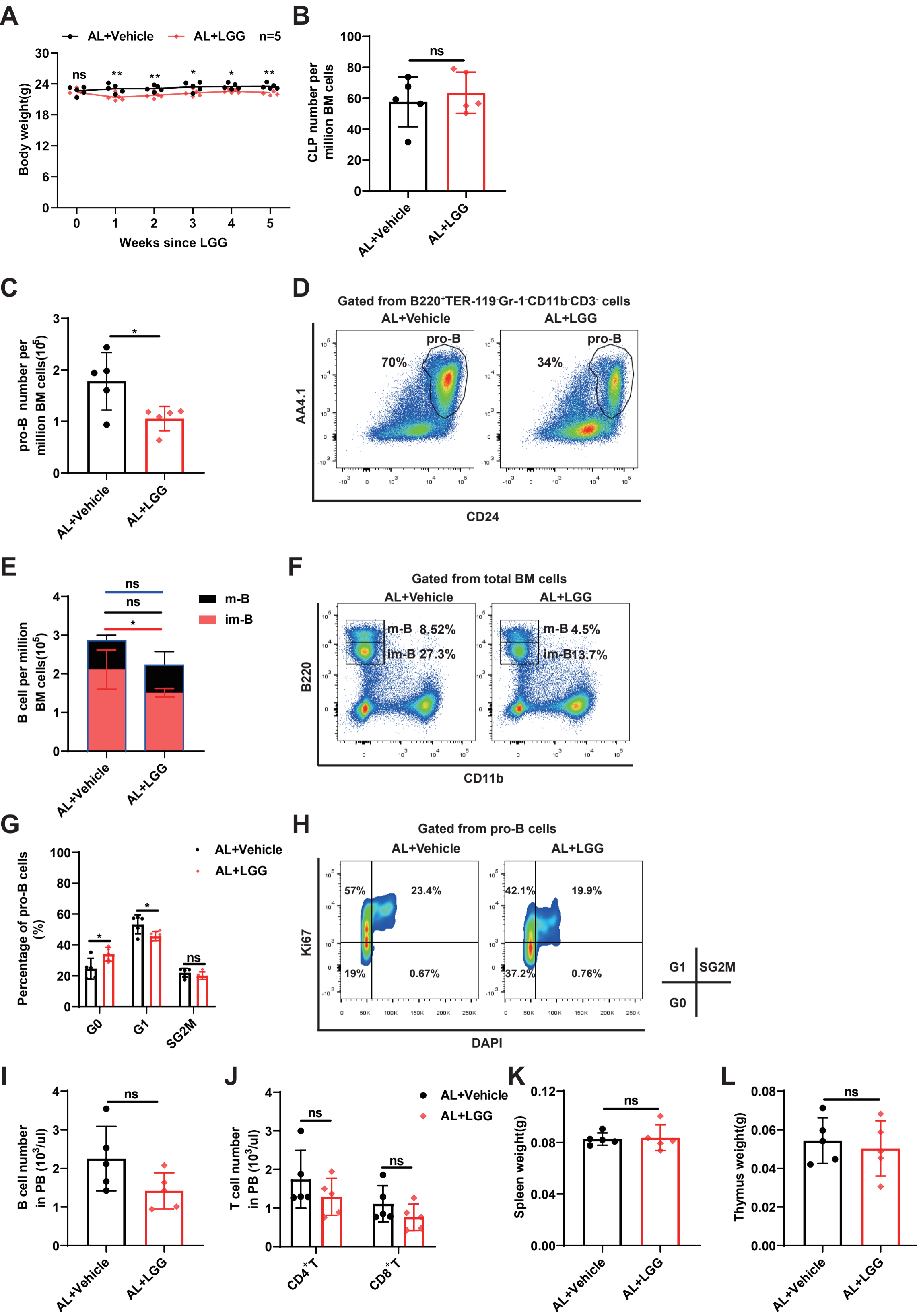

Tao and Wang et al\_Supplementary Figure 1

Supplement: Supplemental Material [file KGMI_A_2117509_SM3008.zip › Tao and Wang et al_Supplementary Figure 1.pdf]

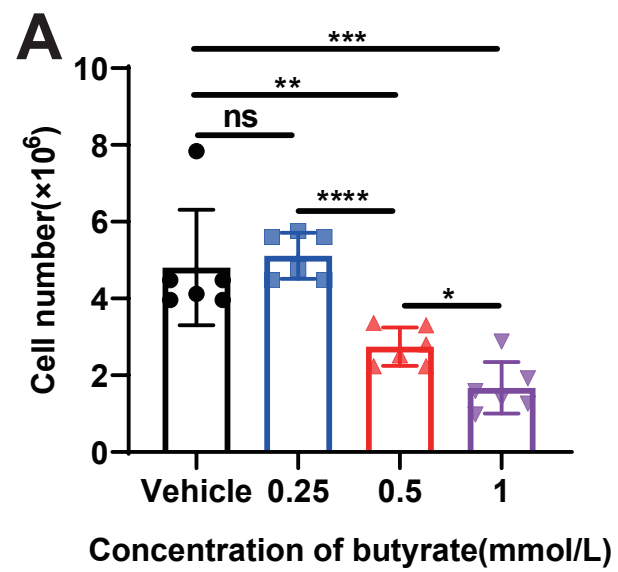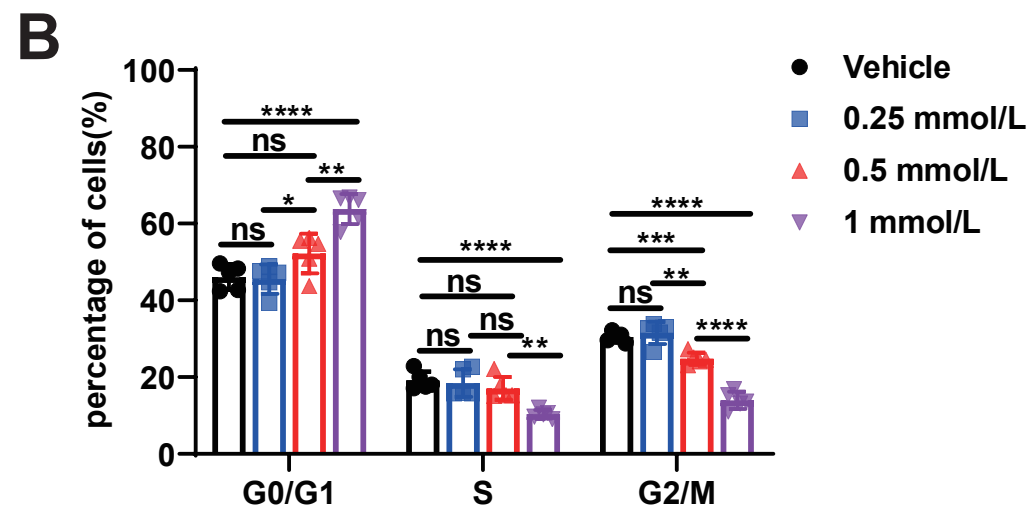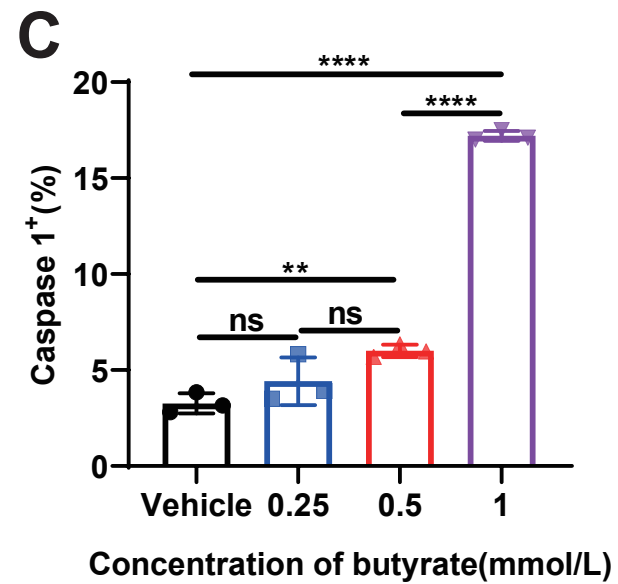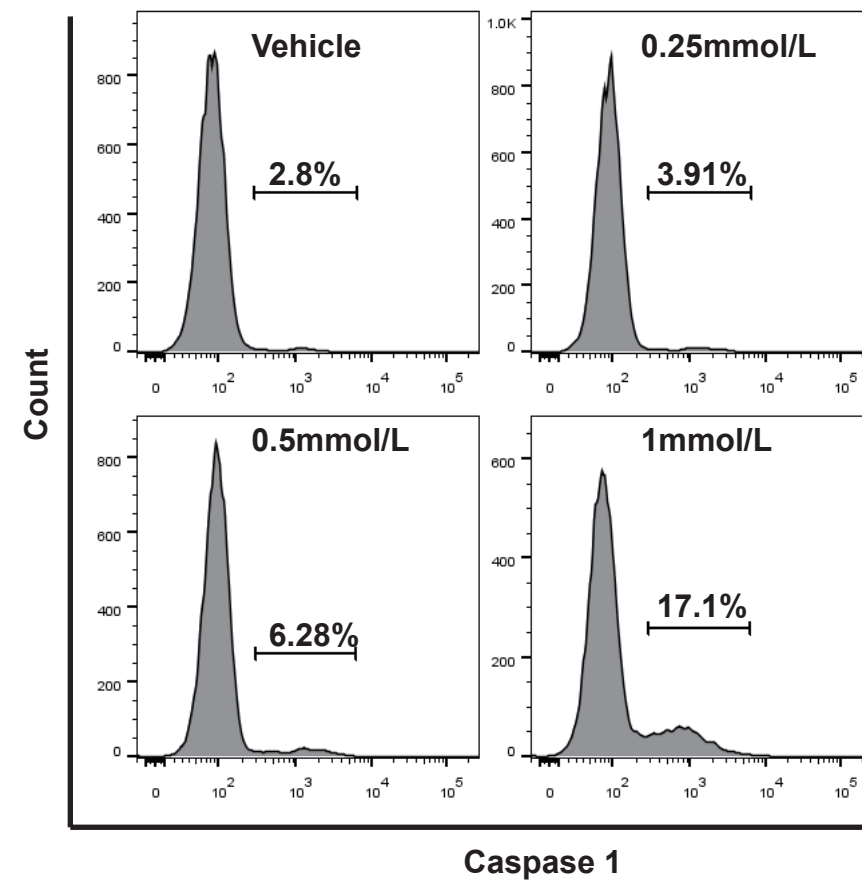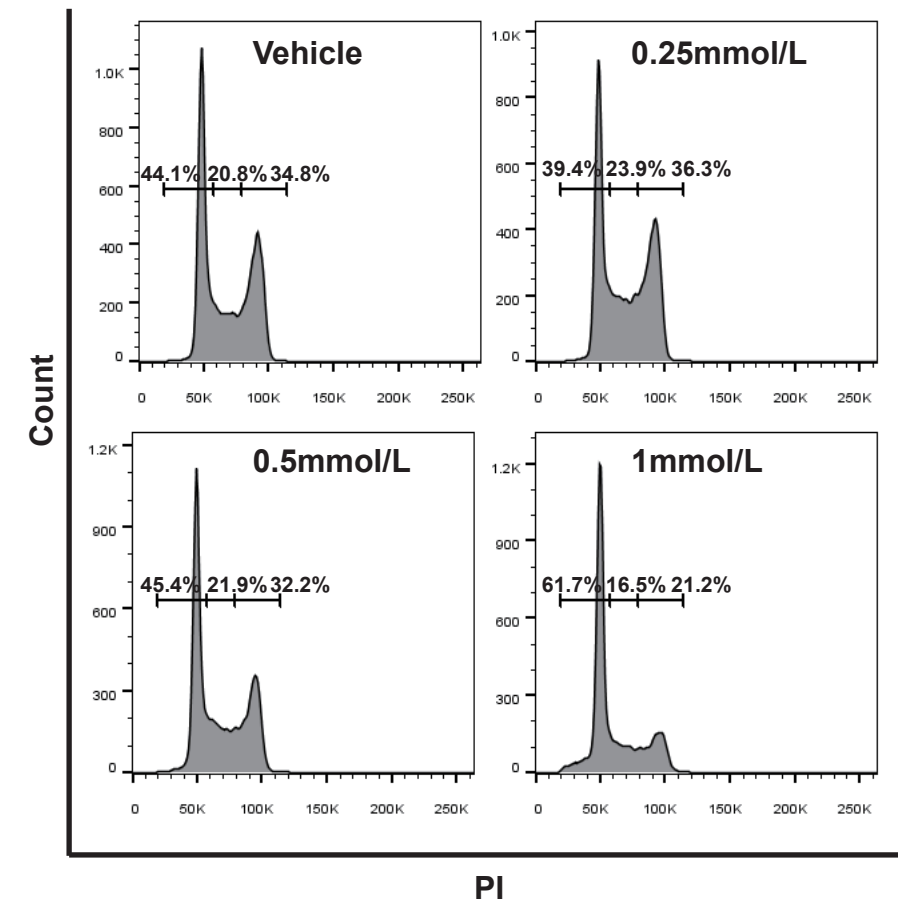

Tao and Wang et al\_Supplementary Figure 2

Supplement: Supplemental Material [file KGMI_A_2117509_SM3008.zip › Tao and Wang et al_Supplementary Figure 2.pdf]
